# Retrospective analysis of 264 multiple myeloma patients

**DOI:** 10.3892/ol.2012.1018

**Published:** 2012-11-09

**Authors:** CHUANYING GENG, NIAN LIU, GUANGZHONG YANG, AIJUN LIU, YUN LENG, HUIJUAN WANG, LIHONG LI, YIN WU, YANCHEN LI, WENMING CHEN

**Affiliations:** Department of Hematology, Beijing Chaoyang Hospital, Capital Medical University, Beijing 100020, P.R. China

**Keywords:** multiple myeloma, autologous stem cell transplantation, bortezomib, survival

## Abstract

Multiple myeloma (MM) is the second most common hematological malignancy in China. However, there are only a small number of large cohort studies demonstrating the clinical features of newly diagnosed MM. In the present study, 264 newly diagnosed MM patients from the Beijing Chaoyang Hospital were retrospectively analyzed. The median patient age was 59 years (range, 28–84) and the most common monoclonal protein (42%) was the IgG subtype. Of the 49 patients detected by FISH, 10.2, 2.0 and 12.2% demonstrated del(17p), t(14;16) and t(4;14), respectively. In total, 228 (86%) patients achieved either a complete response (CR), a very good partial response (VGPR) or a partial response (PR). The overall response rate (ORR) in non-autologous stem cell transplantation (non-ASCT) patients was 83.0%, with 48 (18.2%), 7 (2.7%) and 121 (45.8%) patients achieving CR, VGPR and PR, respectively. ASCT patients achieved at least a PR prior to ASCT, and ASCT was not able to increase the ORR (P=0.55). Non-ASCT patients who received bortezomib-based regimens demonstrated an improved ORR compared with those who received regimens that did not contain bortezomib (92.3% vs. 75.8%; P<0.05). With a median follow-up time of 20 months, the estimated median progression-free survival (PFS) and overall survival (OS) times were 27.6 and 61.0 months, respectively. The OS time of patients with high-risk cytogenetic abnormality, del(17p), t(14;16) and t(4;14), was shorter compared with that of other patients (30.2 months vs. not reached, P=0.029). Patients who achieved a CR/VGPR in the ASCT group demonstrated a greater OS time compared with non-ASCT patients (P=0.031). Relapsed patients who received bortezomib-based regimens did not demonstrate a longer survival time post-relapse compared with those who received non-bortezomib-based regimens (26.5 months vs. 10.5 months; P=0.271). The current study presented the clinical characteristics of MM patients who were initially treated at the Beijing Chaoyang Hospital. Bortezomib-based regimens and ASCT were able to improve the OS of MM patients.

## Introduction

Multiple myeloma (MM) is a plasma-cell neoplasm that is characterized by skeletal destruction, renal failure, anemia, and hypercalcemia (1–4). The most common symptoms on presentation are fatigue, bone pain and recurrent infection. MM is the second most common hematological malignancy after non-Hodgkin's lymphoma, and the incidence of MM in China is ∼1/100,000 (1). The median survival time following diagnosis is ∼3 years.

As a first-line treatment, melphalan with prednisone (MP) have remained the gold standard of therapy for a number of decades. At present, high-dose therapy with autologous stem cell transplantation (ASCT) has been demonstrated to improve progression-free survival (PFS) and/or overall survival (OS) in patients <65 years of age. MP-thalidomide and MP-bortezomib have become standard therapies for patients who are non-transplant candidates. The purpose of the present study was to analyze the clinical characteristics and outcomes of MM patients who were initially diagnosed and treated in our hospital.

## Patients and methods

### Patients

A total of 264 MM patients who fulfilled the International Myeloma Working Group (IMWG) criteria at the Beijing Chaoyang hospital in China were enrolled from January 1, 2006 to December 31, 2011. In accordance with the protocol approved by the Medical Ethics Committee at the Beijing Chaoyang Hospital, retrospective analyses were carried out on the patients' data. All 264 MM patients were initially diagnosed and received therapy at the Beijing Chaoyang Hospital. Physical examinations, image diagnostic and laboratory tests, bone marrow aspirates and biopsies were conducted to evaluate the disease condition of patients. Data collected included age, gender, Durie Salmon (DS) and International Staging System (ISS) stages, date of diagnosis, cytogenetic abnormality, serum creatinine value, disease progression information and survival status. All patients provided written, informed consent prior to chemotherapy and ASCT.

### Therapy and assessments

Induction therapy was performed following patient diagnosis. The first-line induction regimens included thalidomide and dexamethasone (TD); melphalan, prednisone and thalidomide (MPT); thalidomide, amycin and dexamethasone (TAD); vincristine, amycin and dexamethasone (VAD); vincristine, amycin, dexamethasone and thalidomide (VADT); bortezomib and dexamethasone (BD); bortezomib, thalidomide and dexamethasone (BTD); bortezomib, epirubicin and dexamethasone (PAD) and bortezomib, epirubicin, dexamethasone and thalidomide (PADT). Of the 264 patients, 212 (80.3%) patients did not receive ASCT and 52 (19.7%) patients received ASCT after achieving remission. Thalidomide (100 mg/day) was adminsitered to 89.6% patients as maintenance therapy, while other patients received interferon as maintenance therapy due to the presence of peripheral neuropathy and constipation.

Responses were assessed according to the IMWG uniform response criteria (5). Response criteria included a complete response (CR), a very good partial response (VGPR), a partial response (PR) and progressive disease (PD). PFS time was measured as the time period from the start of treatment to disease progression or mortality. OS time was defined as the time period from the initial diagnosis to mortality by any cause.

### Statistical analysis

Statistical analyses were performed using the Statistical Package for the Social Sciences (SPSS) 15.0 software (SPSS Inc., Chicago, IL, USA). PFS and OS were analyzed with the Kaplan-Meier method. A log-rank test was utilized to assess the differences between subgroups. P<0.05 was considered to indicate a statistically significant difference.

## Results

### Clinical features

Of the 264 patients with MM, 146 (55.3%) were male and 118 (44.7%) were female. Table I shows the characteristics of the 264 patients prior to treatment. The median patient age was 59 years (range, 28–84). The majority of patients were ≤65 years (78.4%). The most common monoclonal protein identified in myeloma was the IgG type (42%). Additionally, 225 (85.2%) patients presented with DS stage III, while 145 (54.9%) patients presented with ISS stage III. Renal insufficiency was demonstrated in 28.0% of patients. High-risk cytogenetic abnormality was detected in 49 patients using fluorescence *in situ* hybridization (FISH). The FISH markers included t(4;14), t(14;16) and del(17p), and were present in 12.2, 2.0 and 10.2% patients, respectively.

### Efficacy

**Figure 1.**
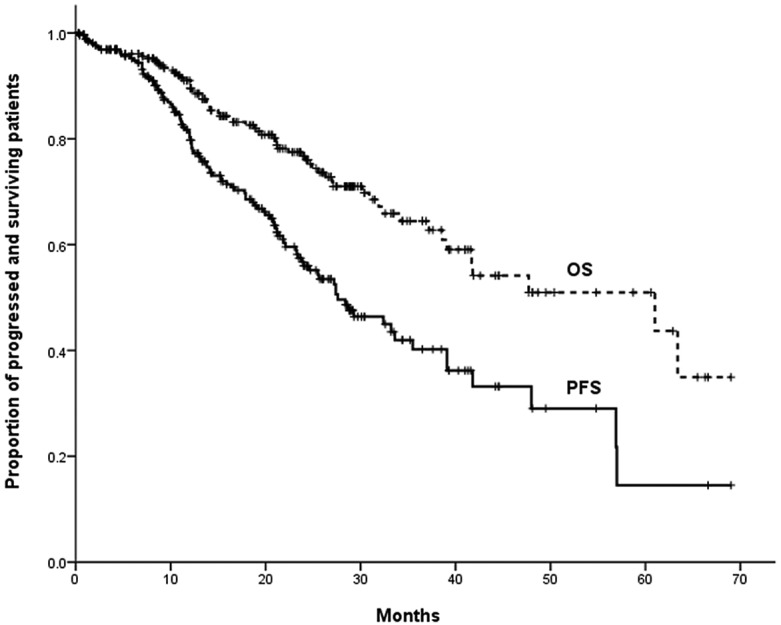
Kaplan-Meier plot of the progression-free survival (PFS) and overall survival (OS) in 264 patients. Kaplan-Meier analysis demonstrated that the estimated median PFS and OS times were 37.6 and 61.0 months, respectively, while the median follow-up time was 20 months for the 264 patients.

A CR, VGPR or PR was achieved in 228/264 (86%) patients. With a median follow-up time of 20 months for all patients, the estimated median PFS time was 37.6 months and the estimated median OS time was 61.0 months (Fig. 1). Of the 52 patients who received ASCT, CR, VGPR and PR were achieved prior to ASCT in 28 (53.9%), 9 (17.3%) and 15 (28.9%) patients, respectively, and post-ASCT in 31 (59.6%), 7 (13.5%) and 14 (26.9%) patients, respectively. The CR rate was not significantly higher post-ASCT compared with prior to ASCT (P=0.55) (Table II). In the non-ASCT group, the overall response rate (ORR) was 83.0%, with 48 (18.2%), 7 (2.7%) and 121 (45.8%) patients achieving a CR, VGPR and PR, respectively.

Of the 212 non-ASCT patients, 120 (56.6%) patients received induction regimens without bortezomib and 92 (43.4%) patients received bortezomib-based regimens. Among the non-ASCT groups, patients who received bortezomib-based regimens demonstrated a greater ORR compared with those who did not receive bortezomib (92.3% vs. 75.8%; P<0.05) (Table III).

Among 155 patients aged ≤65 years, the 71 (45.8%) patients who received bortezomib-based regimens demonstrated a greater ORR compared with the 84 (54.2%) patients who received regimens without bortezomib (93.0% vs. 76.2%, P<0.05). Bortezomib-based regimens, as opposed to regimens without bortezomib, also demonstrated a greater ORR in patients >65 years (81.0 vs. 75%; P<0.05) (Table III).

### Subgroup analysis of PFS

**Figure 2.**
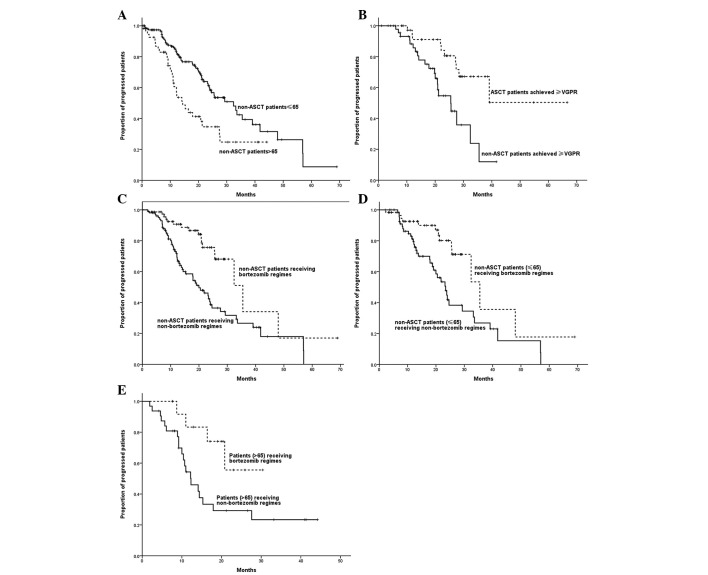
Kaplan-Meier plot of progression-free survival (PFS) time in various groups. (A) The PFS time of patients >65 years is shorter than that of patients ≤65 years (P= 0.001). (B) For CR/VGPR patients who received ASCT, the PFS time is longer than that of patients who did not receive ASCT (P=0.002). (C) Non-ASCT patients who received bortezomib-based regimens demonstrate a longer PFS time compared with those who received regimens without bortezomib (P=0.001). (D) Patients ≤65 years who received bortezomib-based regimens demonstrate a longer PFS time compared with those who received regimens without bortezomib (P=0.003). (E) Bortezomib-based regimens, as opposed to regimens without bortezomib, also lead to a longer PFS in patients >65 years (P=0.026).

A significant correlation was observed between age and PFS time, with the PFS time of patients >65 years being shorter than that of patients ≤65 years (14.4 vs. 32.4 months; P=0.001). With a median follow-up time of 20 months, for patients who achieved CR/VGPR following induction therapy, the estimated median PFS time of patients who received ASCT was longer than that of patients who did not receive ASCT (not reached vs. 35.5 months; P=0.002). Non-ASCT patients who received bortezomib-based regimens demonstrated a longer PFS time compared with those who received regimens without bortezomib (37.0 vs. 25.6 months; P= 0.001). Among patients ≤65 years, those who received bortezomib-based regimens demonstrated a longer PFS time than those who received regimens without bortezomib (35.5 vs. 23.4 months; P=0.003). Bortezomib-based regimens also led to a longer PFS time in patients >65 years (not reached vs. 12.3 months; P=0.026) (Fig. 2).

### Subgroup analysis of OS time

**Figure 3.**
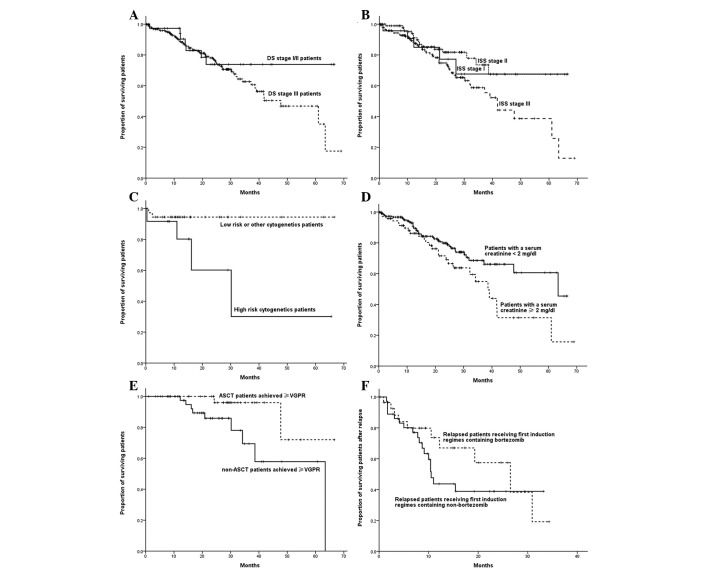
Kaplan-Meier plot of overall survival (OS) time in the various groups. (A and B) There is no statistical significance in OS time between DS stage (P=0.209) and ISS stage (P=0.051). (C) High-risk cytogenetic abnormality, del(17p), t(14; 16) and t(4; 14), is a poor prognostic factor (P=0.029). (D) High baseline serum creatinine (≥2 mg/dl) is a poor prognostic factor (P=0.025). (E) CR/VGPR patients who received ASCT demonstrate a longer OS time than that of patients who did not receive ASCT (P=0.031). (F) Relapsed patients who received regimens containing bortezomib in the initial stages of treatment demonstrate the same median survival time post-relapse as those who received regimens that did not contain bortezomib (P=0.271).

The OS time for DS stage III was shorter than that of DS stage I or II patients, although no significant difference was observed (P=0.209). No significant difference was detected in the estimated median OS time among patients with ISS stage I, II or III (not reached vs. not reached vs. 48.0 months, respectively; P=0.051). The OS time of patients with high-risk cytogenetic abnormality, del(17p), t(14; 16) and t(4; 14), was significantly shorter compared with that of other patients (30.2 months vs. not reached; P=0.029). Patients who had a baseline serum creatinine level <2 mg/dl demonstrated a greater OS time compared with that of patients with a baseline serum creatinine level ≥2 mg/dl (63.4 vs. 38.6 months; P=0.025). Among all patients who achieved a CR/VGPR, those who received ASCT demonstrated a greater OS time compared with non-ASCT patients (not reached vs. 63.4 months; P=0.031). In relapsed patients, those who received regimens containing bortezomib in the initial treatment phase demonstrated the same median survival time following relapse compared with those who received regimens that did not contain bortezomib (26.5 vs. 10.5 months; P=0.271) (Fig. 3).

**Figure 4.**
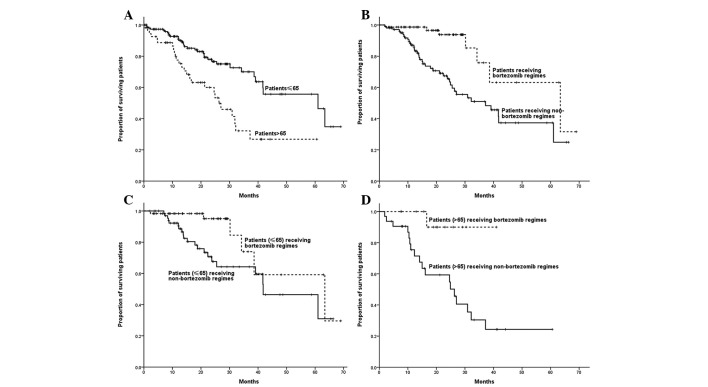
Kaplan-Meier plot of overall survival (OS) in non-ASCT patients. (A) The OS time of patients >65 years is shorter than that of patients ≤65 years (P=0.001). (B) Non-ASCT patients who received bortezomib-based regimens demonstrate a longer OS time compared with those who received regimens without bortezomib (P= 0.001). (C) Patients ≤65 years who received bortezomib-based regimens demonstrate a longer OS time compared with those who received regimens without bortezomib (P=0.021). (D) Bortezomib-based regimens, as opposed to regimens without bortezomib, also demonstrate a longer OS time in patients >65 years (P=0.017).

Among non-ASCT patients, age was correlated with OS time, with the OS time of patients >65 years being shorter than that of patients ≤65 years (26.3 vs. 61.0 months; P=0.001). The OS time of patients who received bortezomib-containing regimens was significantly longer than that of patients who received regimens without bortezomib (63.4 vs. 37.2 months; P=0.001). Patients ≤65 years who received bortezomib-based regimens demonstrated a longer OS compared with those who received regimens without bortezomib (63.4 vs. 41.8 months; P=0.021). Bortezomib-based regimens, as opposed to regimens without bortezomib, led to a longer OS time in patients >65 years (not reached vs. 26.3 months; P=0.017) (Fig. 4).

## Discussion

The present study retrospectively analysed the clinical data of 264 MM patients at the Beijing Chaoyang Hospital in China. The data revealed that MM frequently occurred in the elderly population. In the majority of western countries, the median age of patients with MM is ∼65 years. However, in the present study, the median age was only 59 years. One possibility is that ethnic diversity may account for the difference. However, the median age of MM patients may have increased as the Chinese life expectancy has steadily increased. Basic investigations are required to identify the reason for the difference. In this study, the majority of patients were in either DS or ISS stage III, which differed from other studies. This difference reinforced the requirement for greater medical attention and higher sensitivity tests to identify more patients in early stage. In this study, the most common monoclonal protein was also IgG concordant with a study (4). Moreover, the prevalence of renal insufficiency was 28%, which is in accordance with the majority of studies. Three common high-risk cytogenetic abnormalities, t(4;14), t(14;16) and del(17p), were detected by FISH in 49 patients; the occurences demonstrated were similar to those of other studies.s (6–7).

A key finding of this study was that patients >65 years demonstrated an inferior outcome compared with non-ASCT patients ≤65 years, as measured by the OS time. The reason for the reduced efficacy in elderly patients is likely to be multifactorial. Elderly patients may have been less heavily pretreated with respect to prior therapy, and patients >65 years did not undergo ASCT. Herein, we excluded the function of ASCT; however, age remained an influence on patient survival. It may be that older patients and physicians were less accepting of high-dose chemotherapy. Thus, one possibility for the poorer outcome in elderly patients is the lower average daily dose that older patients received. Another possible explanation for the age effect is that the biology of malignant plasma cells and the bone marrow microenvironment may be different in older, compared to younger, patients. There are conflicting studies concerning the impact of patient age on prognosis in newly diagnosed MM. Certain studies have demonstrated that elderly patients have an inferior survival compared with younger patients, whereas others have reported no effect of age on survival (8).

Although the DS system has previously been demonstrated to be an effective staging tool for patients with multiple myeloma, a number of studies have revealed that it is not capable of indicating a significant survival difference between stages I, II, and III (9). As only 1% of patients in the current study were in DS stage I, DS stages I and II were combined, and compared with DS stage III. A similar result was found in that DS stages I/II and III were not able to significantly differentiate patients, although the OS time for DS stage III was shorter than that of DS stage I/II patients. A combination of serum β2-microglobulin and serum albumin provided the simplest, most powerful and reproducible three-stage classification, i.e., the ISS staging system (10). No difference in the median OS time of patients with ISS stage I, II or III was observed. Therefore, our study was not able to confirm the prognostic utility. It is possible that novel drugs modified the prognostic value of the ISS stage. Up to 20% of newly diagnosed MM patients demonstrated renal impairment complications. Renal insufficiency, in particular dialysis dependency, was an independent poor prognostic factor in MM, while the majority of patients were unable to achieve dialysis independence. Renal impairment (serum creatinine level ≥2 mg/dl) and a poor OS time were evident in 28% of MM patients, whereas renal impairment was a poor prognostic factor.

Cytogenetic status was the most important prognostic factor in patients with MM. Patients with hyperdiploidy or immunoglobulin heavy chain (IgH) translocation t(11;14) demonstrated good or average survival times, respectively. Poor cytogenetic features were: 17p deletion, chromo-some 1q gains, t(4;14) and t(14;16)(11–12). In this study, three common high-risk cytogenetic abnormalities were detected in 49 patients by FISH. The results demonstrated that patients with high-risk cytogenetic abnormalities had a poor OS time compared with other patients.

High-dose melphalan with autologous stem cell support has been an integral part of myeloma therapy, either as salvage therapy or as consolidation of an initial remission. The response to therapy is a crucial prognostic factor in patients with MM. Patients who achieve CR demonstrated an event-free survival (EFS) and an OS time significantly longer than those who remained in the PR stage. Improvement in the depth of response has been associated with a significantly longer EFS and OS time (13–15). In this study, ASCT was not able to significantly increase the CR rate of patients. It is possible that the majority of patients had achieved at least a VGPR prior to ASCT and we were not able to detect the clearing effect of ASCT on minimal residual disease. In order to exclude the influence of the response to therapy on PFS and OS times, we compared the PFS and OS times of ASCT patients who achieved CR/VGPR prior to ASCT with those of the non-ASCT patients who also achieved CR/VGPR. ASCT was able to improve the PFS (P=0.002) and OS (P=0.031) times. High-dose melphalan with autologous stem cell support was able to benefit MM patients.

The boronic dipeptide, bortezomib, targets the proteasome to prevent intracellular protein degradation (16–17). It causes cell cycle arrest, anti-angiogenic effects, induction of the stress response and apoptosis of multiple myeloma cells mediated by caspase-8/9. In the present study, patients who received bortezomib-containing regimens had greater PFS and OS times. Among non-ASCT patients, the OS time of patients receiving bortezomib-containing regimens was significantly longer than that of patients receiving regimens without bortezomib (P=0.001). Among non-ASCT patients, both ≤65 and >65 years, patients who received bortezomib-based regimens demonstrated a longer OS time compared with those who received regimens without bortezomib. In relapsed patients, those who received regimens containing bortezomib in the initial stages of treatment did not demonstrate an increased median survival time post-relapse compared with those who did not receive regimens containing bortezomib (P=0.271). However, the number of relapsed patients was limited, and significant differences in OS time may be observed with an increased sample size. These results demonstrated that bortezomib is effective in MM patients.

This study presented the clinical characteristics of MM patients who were initially diagnosed and received treatment at the Beijing Chaoyang Hospital, and the effects of the majority of common regimens on newly diagnosed patients. Age was demonstrated to influence the OS of patients. Neither the DS nor the ISS stage were able to provide an exact prognosis, while renal function insufficiency was a poor prognostic factor and high risk cytogenetic abnormality indicated a poor prognosis. ASCT and bortezomib-based regimens were able to prolong PFS and OS times.

## Figures and Tables

**Table I.** Characteristics of 264 newly diagnosed MM patients.

Characteristic	Values
Age (years, median (range))	59 (28–84)
≤65 (median) (n, %)	56 (207, 78.4%)
>65 (median) (n, %)	72 (57, 21.6%)
Gender	
Male	146, 55.3%
Female	118, 44.7%
M^a^ component	
IgA	52, 19.7%
IgD	21, 8.0%
IgG	111, 42.0%
Non-secretory	13, 4.9%
K	33, 12.5%
λ	34, 12.9%
Durie-Salmon stage	
I	3, 1.1%
II	36, 13.6%
III	225, 85.2%
ISS stage	
I	23, 8.7%
II	96, 36.4%
III	145, 54.9%
Renal function	
A (<2 mg/dl serum creatinine)	190, 72.0%
B (≥2 mg/dl serum creatinine)	74, 28.0%
FISH	
del(17p)	5/49, 10.2%
t(14;16)	1/49, 2.0%
t(4;14)	6/49, 12.2.%

a Myeloma. MM, multiple myeloma; ISS, International Staging System.

**Table II.** Response rates of 52 patients with MM before and after ASCT.

	No. of patients (%)			
Response	CR	VGPR	PR	SD or PD
Prior-ASCT	28 (53.85)	9 (17.31)	15 (28.85)	0 (0)
Post-ASCT	31 (59.62)	7 (13.46)	14 (26.92)	0 (0)

MM, multiple myeloma; ASCT, autologous stem cell transplantation; CR, complete response; VGPR, very good partial response; PR, partial response; SD, stable disease; PD, progressive disease.

**Table III.** Effects of regimens and age on the response rate of non-ASCT patients.

	Response (%)		
Variable	CR	VGPR or PR	SD or PD
Regimen			
Without bortezomib	11.67	64.17	24.17
With bortezomib	36.96	55.43	7.61
Age (years)			
≤65	23.23	61.94	14.84
>65	21.05	56.14	22.81
≤65 years			
Without bortezomib	11.90	64.29	23.81
With bortezomib	36.62	59.15	4.23
>65 years			
Without bortezomib	11.11	63.89	25.00
With bortezomib	38.10	42.86	19.05

ASCT, autologous stem cell transplantation; CR, complete response; VGPR, very good partial response; PR, partial response; SD, stable disease; PD, progressive disease.
